# Acceptance of Men Living With HIV Toward Treatment-Supportive Mobile Apps Using the Unified Theory of Acceptance and Use of Technology: Cross-Sectional Study

**DOI:** 10.2196/83065

**Published:** 2026-02-10

**Authors:** Fabian Kempen, Ranujan Chandrakumar, Stefan Esser, Lisa Maria Jahre, Martin Teufel, Alexander Bäuerle

**Affiliations:** 1 Clinic for Psychosomatic Medicine and Psychotherapy, LVR-University Hospital, University of Duisburg-Essen, Virchowstr., 174, Essen, 45147, Germany, +49 201 43 87 55 2; 2 Department of Dermatology, University Hospital Essen, University of Duisburg-Essen, Essen, Germany; 3 Center for Translational Neuro- and Behavioral Sciences (C-TNBS), University of Duisburg-Essen, Essen, Germany

**Keywords:** HIV, eHealth, mobile health, mHealth, mobile app, people living with HIV, telemedicine, Unified Theory of Acceptance and Use of Technology, UTAUT

## Abstract

**Background:**

Despite a 40-year prevalence of HIV, the AIDS epidemic prevails. Effective AIDS treatment requires specialist care and high adherence often hindered by structural issues in care access. Innovative eHealth solutions like treatment-supportive mobile apps can help address these issues. Successful implementation depends on user acceptance. Acceptance can be operationalized as behavioral intention and measured through the Unified Theory of Acceptance and Use of Technology (UTAUT).

**Objective:**

This study examines the acceptance and its influencing factors of treatment-supportive mobile apps among men living with HIV.

**Methods:**

A cross-sectional study was conducted among 172 men living with HIV between September 2021 and April 2024. In addition to the collection of sociodemographic, medical, and eHealth-related data, acceptance and its influencing factors were assessed by applying the UTAUT model. A multiple hierarchical regression analysis was conducted.

**Results:**

High acceptance of treatment-supportive mobile apps in men living with HIV was reported by 45.3% (n=78) of the participants. Significant predictors of acceptance were age (β=−0.27; *P*<.001); health literacy regarding disease (β=0.11; *P*<.001); eHealth literacy (β=0*.*10; *P*=.001); internet anxiety (β=−0*.*18; *P*=.04); and the UTAUT predictors: effort expectancy (β=0*.*38; *P*<.001), performance expectancy (β=0*.*24; *P*<.001), and social influence (β*=*0.40; *P*<.001). The UTAUT model explained 72% of the variance in acceptance.

**Conclusions:**

Since the acceptance of eHealth services is a reliable indicator of the actual usage behavior, the results of this study are a promising basis for the successful implementation of eHealth offerings in the group of men living with HIV.

## Introduction

### Background

Worldwide, 37.7 million people are infected with HIV [1]. Current statistical records show that HIV infection continues to be a globally significant disease. In Germany, the number of people living with HIV was approximately 97,000 in 2020 [2]. Complete elimination of the virus from the human body is not possible due to latent infection of cells, which have a long survival time in the body [3].

The supply of care for people living with HIV is fraught with several problems. Access to medical care is gradually deteriorating. Comprehensive medical coverage is difficult to provide for an aging population, as obstacles arise due to high costs and distances between patient and health care provider to be bridged [4]. In general, a trend toward urban oversupply in treatment options and rural undersupply becomes apparent [5,6].

Highly active antiretroviral therapy, a combination of several antiviral drugs that inhibit the replication of HIV, raises the life expectancy of people living with HIV to almost the same level as the general population if the therapy is effective [7,8]. The prescribed substances can have a variety of side effects, such as lipoatrophy, fat distribution disorders, liver dysfunction [9], or weight gain [10]. Nonadherence or inconsistent intake leads to disease progression in the vast majority of patients [11-13]. Previous work has shown that nonadherence can be caused by multiple factors such as forgetting to take medication or changes in daily routine [14]. Moreover, mental disorders are positively related to low adherence [15,16]. The prevalence of mental disorders [17,18] and incidences of suicides [19] in people living with HIV are higher compared to the general population.

eHealth describes applications that use the possibilities offered by modern information and communication technologies to support the treatment and care of patients. eHealth and mobile health (mHealth) are related concepts, with eHealth encompassing the broader use of digital technologies in health care, while mHealth refers specifically to the use of mobile devices to support medical and public health practice [20]. In terms of health-related outcomes in many mental and somatic disorders, eHealth interventions are effective options to support treatment [21-23]. Moreover, eHealth services are often less expensive and more flexible regarding time and location [24]. Medical care problems of people living with HIV are largely structural, stemming from systemic issues like stigma, limited health care access, and lack of integrated services [25]. Regarding the needs of people living with HIV, therefore, the implementation of eHealth technologies offers several advantages: they ensure care beyond direct contact with professionals [26], are less time-consuming and transport-intensive, and offer more flexibility [27]. Furthermore, they offer more privacy by avoiding social stigma and providing the possibility to discuss sensitive issues in a familiar, secure environment [28,29]. In addition, a study about a mobile app that allows virtual visitation of patients demonstrated that medical apps can greatly simplify patient contact [30].

The COVID-19 pandemic has opened the door to a groundbreaking revolution in eHealth services and has highlighted the importance of these innovative tools. The pandemic offered a unique opportunity to further develop and establish eHealth applications for people living with HIV [31].

Despite the aforementioned benefits, the implementation of eHealth applications in routine care has proven to be difficult [32]. Essential for the implementation of eHealth technologies is the target groups’ intention to use them in the first place [33]. The acceptance of these offers can also be operationalized as intention to use such technologies [34], which makes acceptance a significant prerequisite for the successful implementation of eHealth technologies. Studies that were previously conducted to assess the acceptance of eHealth technologies among people living with HIV yielded mixed results: in an online multicenter study from France, 30.8% of respondents were eHealth skeptics and 69.2% were supporters [35]. Another study in two HIV clinics that provided telemedicine consultation during the pandemic found that about 58% of participants did not find the offer useful [27]. The spectrum of eHealth offerings is vast, so more complex assessments on influencing factors of acceptance are needed.

As men represent a significant proportion of the HIV-positive population, it is particularly important to assess their acceptance to eHealth applications. In many regions, men who have sex with men are infected with HIV at a higher rate than average rate compared to the rest of the population [36]. This group not only has a higher risk of HIV, but also tends to be underserved in traditional health care systems, as studies have shown that men are less likely to seek regular medical care [37].

These structural barriers result in structural deficits and undersupply among men living with HIV, which could be addressed effectively with eHealth technologies by offering flexible, low-threshold, and stigma-reduced options for care. Examining how well these technologies are accepted by men living with HIV is therefore essential for their effective implementation and for assessing their potential impact.

Previous studies have applied theoretical models to measure the acceptance of health technologies. The technology acceptance model [38] assumes that perceived usefulness and perceived ease of use determine the intention to use a technology. The Unified Theory of Acceptance and Use of Technology (UTAUT) [34] extends the technology acceptance model by integrating constructs from 8 acceptance models, including social influence and facilitating conditions. It states that acceptance is mainly influenced by the 3 core predictors: performance expectancy (PE), effort expectancy (EE), and social influence (SI) [34]. PE stands for the extent to which an individual believes he or she will benefit from using a technology. EE refers to the amount of effort an individual believes he or she has to expend to use a technology. SI describes the extent to which an individual believes that important people, such as friends or family, would approve of the use of a technology. Acceptance itself is operationalized as behavioral intention (BI) as the fourth parameter in the model. The UTAUT model has proven superior in predicting the BIs. The model has also been validated in the context of eHealth technologies [39] and has been applied multiple times in different samples [40-48] including studies focusing on men who have sex with men [49]. However, there is currently no validated assessment of the acceptance of eHealth technologies and its influencing factors among men living with HIV.

### Objectives

The well-established and validated UTAUT model is applied and extended to assess possible influencing factors of acceptance beyond its 3 core predictors. While the original UTAUT model includes the 3 core predictors: PE, EE, and SI, the expanded UTAUT model additionally considers sociodemographic, medical, and eHealth-related factors. These include age, educational level, employment status, HIV-specific clinical markers, and previous experience with eHealth. It has been shown that the acceptance of digital health services is also influenced by structural, health-related, and technological factors [50,51]; therefore, the extended UTAUT model was applied in this study. By including additional parameters, we expect to capture a broader range of barriers and facilitators to the acceptance of telemedicine services, thereby reflecting the complexity of health care use among people with chronic conditions such as HIV.

This study aims to investigate the acceptance of treatment-supporting mobile apps among men with HIV using an extended version of UTAUT. In particular, the study has three objectives as follows:

1. To assess the general acceptance level of treatment-supporting mHealth apps in this population group.
2. To assess the extent to which the three core indicators of UTAUT (PE, EE, and SI) can predict acceptance.
3. To determine the additional contribution of sample-related factors, including sociodemographic characteristics, medical variables, health literacy, eHealth literacy, and internet anxiety, by explaining variations in acceptance beyond the core constructs of UTAUT.

By integrating these expanded predictors into the UTAUT framework, the study aims to provide a more comprehensive understanding of the determinants that influence adoption and support the targeted development of the user-centered digital health interventions for men living with HIV.

## Methods

### Ethical Considerations

The study was conducted in accordance with the Declaration of Helsinki and was approved by the ethics committee of the Medical Faculty of the University of Duisburg-Essen (19-89-47-BO). Electronic informed consent was mandatory to participate. Participation in the study was voluntary, data were anonymous, and no compensation was provided.

### Study Design, Participants, and Procedure

A cross-sectional online-based survey study was conducted to examine the acceptance of treatment-supportive mobile apps in a convenience sample of men living with HIV. Recruitment was carried out via flyers and posters in centers specialized in HIV treatment and bars within the queer community in Germany, as well as posts made on HIV-specific social media channels and online support groups on Facebook. Data collection was conducted between September 2021 and April 2024. Participants with a diagnosed HIV infection, adult age (≤18 y), sufficient German language skills, and internet access were eligible for the study. The survey was conducted using the online platform Unipark [50]. Participation was voluntary and anonymous with no financial compensation being offered. Electronic informed consent was mandatory before starting the survey.

The average execution time of the survey was approximately 18 (SD 13) minutes. Out of 336 participants who initially started the survey, 191 completed the survey, which equals a completion rate of 56.8%. Furthermore, 19 participants were excluded because they did not meet the inclusion criteria (sex: male). Ultimately, 172 participants were included in the final data analysis.

### Assessment Instruments

To address the research questions, the online survey contained items on sociodemographic characteristics, medical, and eHealth-related data. The study questionnaire was designed by experts from the fields of medicine, psychology, and eHealth and is provided in Multimedia Appendix 1. The primary outcome was acceptance, operationalized as BI, which was assessed via an adapted version of the UTAUT model [34].

### Sociodemographic and Medical Data

Sociodemographic data were assessed using self-generated items and included age, sex, marital status, educational as well as occupational status, and place of residence (population size).

Medical history was queried via self-generated items toward duration of disease, hospitalizations, and comorbidities. Duration of disease was assessed by the item “How long have you known you are infected with HIV?” Responses were given in years as whole numbers. Hospitalization was assessed by the item “Have you been hospitalized due to your illness?” Respondents could either agree or disagree with this question. Comorbidities were assessed via the item “Do you have any other diseases besides HIV?” Respondents could choose between the options “no” and other exemplary chronic diseases (eg, hepatitis C or diabetes mellitus). In addition, self-generated items about health literacy regarding disease, therapy satisfaction, and adherence to therapy were added. Health literacy regarding disease was assessed by 6 items (eg,“Do you feel well informed about HIV infection and AIDS?”). Responses were given on a 7-point Likert scale (1=“strongly disagree” to 7=“strongly agree”). Internal consistency was good with Cronbach *α*=0.88. Therapy satisfaction was assessed by 4 items (eg,“When you think of your current HIV therapy, how satisfied are you with the following aspects: type of therapy?”). Responses were given on a 7-point Likert scale (1=“not at all satisfied” to 7=“completely satisfied”). Internal consistency was good with Cronbach *α*=0.82. Adherence to therapy was assessed by one item (“Do you take the medication for your HIV therapy as prescribed by your doctor?”). Responses were given on a 5-point Likert scale (1=“no,” 2=“rather no,” 3=“undecided,” 4=“rather yes,” and 5=“yes”). Physical health, mental health, and quality of life were rated on a scale from 0 to 100, with higher scores indicating higher levels of subjective health and quality of life.

### eHealth Data

Furthermore, eHealth literacy was assessed using the revised German version of the eHealth Literacy Scale [51]. Responses were given on a 5-point Likert scale (1=“strongly disagree” to 5=“strongly agree”). Internal consistency was excellent (Cronbach *α*=0.92). Furthermore, digital overload was assessed with 3 items (eg,“I feel burdened by the constant availability”) [45,48,51,52]. Answers were given on a 5-point Likert-type scale (1=“strongly disagree” to 5=“strongly agree”). Internal consistency was good (Cronbach *α*=0.75). Internet anxiety was also assessed via 3 items (eg,“I am afraid I might make an irreversible mistake when using the Internet”) [47,48,53]. Answers were given on a 5-point Likert-type scale (1=“strongly disagree” to 5=“strongly agree”). Internal consistency was good (Cronbach *α*=0.7).

### UTAUT Predictors and Acceptance

To assess acceptance, operationalized as BI, and its influencing factors, an adapted version of the original UTAUT model was applied [34]. The modified UTAUT model contained 3 items in total, with 4 items assessing BI (eg,“I would like to try an app to support my HIV treatment”), and 3 items each assessing SI (eg,“People close to me would approve of me using an app to support my HIV treatment”), PE (eg,“An app to support HIV therapy could help me improve my personal health”), and EE (eg,“Using an app to support HIV treatment would not be an additional burden for me”). Answers were given on a 5-point Likert scale (1=“strongly disagree” to 5=“strongly agree”). Internal consistency for acceptance and its predictors was excellent (BI: Cronbach *α*=0.91; PE: Cronbach *α*=0.9; SI: Cronbach *α*=0.84; and EE: Cronbach *α*=0.77).

### Statistical Analyses

Statistical analyses were performed using R (version 4.4.0; R Foundation for Statistical Computing) [54]. Sum scores were calculated for the revised German version of the eHealth Literacy Scale. Mean scores were calculated for digital overload, Internet anxiety, health literacy regarding disease, therapy satisfaction, and for the UTAUT scales (BI, EE, PE, and SI). Acceptance was operationalized as BI and was further categorized in accordance with prior research [43,45,47]: scores from 1 to 2.34 indicate low acceptance, scores from 2.35 to 3.67 indicate moderate acceptance, and scores from 3.68 to 5 indicate high acceptance. Descriptive statistics were applied for sociodemographic, medical, and eHealth data. Multiple hierarchical regression analysis was conducted to examine drivers and barriers of acceptance of treatment-supportive mobile apps for men living with HIV. Predictors were included blockwise: (1) sociodemographic data, (2) medical data, (3) eHealth data, and (4) UTAUT predictors. The variance inflation factor was used to verify the absence of multicollinearity (all variance inflation factor values <2.0). Visual inspection of QQ plots of the residuals showed no signs of violations against normality. Therefore, a normal distribution of the residuals was assumed. Scatter plots of the standardized residuals and the adjusted predicted values verified homoscedasticity. The level of significance was set to *α*<0.05 for all tests. Effect sizes were reported according to the *Statistical Power Analysis for the Behavioral Sciences* [55], with values around 0.2, 0.5, and 0.8 indicating small, medium, and large effects, respectively.

## Results

### Study Population

Of the 172 participants, the average age was 43.14 (SD 12.76) years. The youngest participant was aged 19 years, whereas the oldest participant was aged 80 years. On average, participants were diagnosed with HIV for 11.60 (SD 8.97) years. Health literacy regarding disease was high (mean 6.36, SD 0.95; range 1‐7). Participants also reported high therapy satisfaction (mean 6.07, SD 0.99; range 1‐7).

The vast majority (n=169, 98.8%) indicated being adherent to treatment. See Table 1 for a complete description of the study population.

**Table 1.** Sample characteristics (N=172).

Characteristics	Value, n (%)
Marital status	
Single	67 (39)
In a relationship	38 (22.1)
Married	53 (30.8)
Divorced or separated	9 (5.2)
Other	5 (2.9)
Educational status	
No or lower secondary education or other	60 (34.9)
Higher secondary education	25 (14.5)
Higher education entrance qualification	30 (17.4)
University education	57 (33.1)
Occupational status	
Student	5 (2.9)
Nonworking	7 (4.1)
Sick leave	7 (4.1)
Employed	119 (69.2)
Self-employed	10 (5.8)
Retired	20 (11.6)
Other	4 (2.3)
Place of residence (population size)	
Large city (>100,000 residents)	122 (70.9)
Medium-sized city (>20,000 residents)	36 (20.9)
Small town (>5000 residents)	10 (5.8)
Rural area (<5000 residents)	4 (2.3)
Comorbidities	
Hepatitis C	8 (4.7)
Diabetes mellitus	9 (5.2)
Oncological comorbidities	3 (1.7)
Chronic kidney disease	2 (1.2)
Kaposi sarcoma	5 (2.9)
Infectious diseases (eg, esophagitis, pneumonia, or fungal)	6 (3.5)
Hypercholesterolemia	14 (8.1)
Hospitalization	62 (36)

Participants reported mean moderate levels of physical health (70.69, SD 19.96; range 0‐100), mental health (70.85, SD 24.46; range 0‐100), and quality of life (74.17, SD 23.89; range 0‐100).

In terms of eHealth, participants reported mean moderate levels of eHealth literacy (22.52, SD 5.80; range 8‐40). Internet anxiety (2.37, SD=1.71; range 1‐10) was low and digital overload was moderate (4.41, SD=2.23; range 1‐10).

### Predictors of Acceptance of Treatment-Supportive Mobile Apps

Acceptance of treatment-supportive mobile apps was moderate (mean 3.44, SD 1.17; range 1‐5). Overall, 45.3% (n=78) of the participants reported high acceptance, 30.2% (n=52) reported moderate acceptance, while 24.4% (n=42) reported low acceptance.

Multiple hierarchical regression analysis was computed to determine predictors of acceptance of treatment-supportive mobile apps in men living with HIV.

In the first step, sociodemographic data were included (*R^2^*=0.131; *R^2^_adj_*=0.09; *F*_7,164_=3.52; *P*=.002). Age (β=−0.27; *P*<.001) was a significant predictor of acceptance. The explained variance of the first step was 13.1%.

In the second step, medical data were included (*R^2^*=0.149; *R^2^_adj_*=0.096; *F*_10,161_=2.82; *P*=.002). This step significantly increased the explained variance to 14.9% (*∆R^2^*=0.18, *F*_3,161_=3.39; *P*<.02). However, no specific medical variable was a significant predictor of acceptance.

In the third step, eHealth data (*R^2^*=0.175; *R^2^_adj_*=0.108, *F*_13,158_=2.59; *P*=.003) significantly increased the explained variance to 17.5% (*∆R^2^*=0.026; *F*_3,158_=4.89; *P*=.003). Internet anxiety (β=−0.18; *P*=.04) was a significant predictor of acceptance.

In the final step, the 3 UTAUT predictors EE, PE, and SI were included (*R^2^*=0.720; *R^2^_adj_*=0.691; *F*_16,155_=24.93; *P*<.001). Explained variance of the final model was significantly increased to 72% (*∆R^2^*=0.545; *F*_3,155_=126.67; *P*<.001). EE (β=0.32; *P*<.001), PE (β=0.21; *P*<.001), and SI (β=0.40; *P*<.001) were significant predictors. Additionally, health literacy regarding disease (β=0.11; *P*<.001) and eHealth literacy (β=0.10; *P*=.04) were significant predictors in this step. Table 2 contains the final UTAUT model of acceptance and its significant predictors. The full hierarchical regression model is provided in Multimedia Appendix 2.

**Table 2.** Hierarchical regression model of acceptance of treatment-supportive mobile apps for men living with HIV (N=172). Only significant predictors of the final model are detailed.

Predictors	B^a^	β^b^	*t* test (*df*)	*R* ^*2*^ ^c^	∆*R*^2^^d^	*P* value
Step 1: sociodemographic data				0.131	0.131	
Step 2: medical data				0.149	0.018	
Health literacy regarding disease	0.13	0.11	−2.08 (155)			.04
Therapy satisfaction	0.12	0.10	1.87 (155)			.06
Step 3: eHealth data				0.175	0.026	
eHealth literacy	0.16	0.10	−2.05 (155)			.04
Step 4: UTAUT^e^ predictors				0.720	0.545	
EE^f^	0.38	0.32	5.28 (155)			<.001
PE^g^	0.23	0.21	3.39 (155)			.001
SI^h^	0.48	0.40	6.60 (155)			<.001

a *B*: unstandardized beta.

b *β*: standardized beta coefficient.

c *R*²: determination coefficient.

d ∆*R*2: changes in *R*2.

e UTAUT: Unified Theory of Acceptance and Use of Technology.

f EE: effort expectancy.

g PE: performance expectancy.

h SI: social influence.

## Discussion

### Principal Findings

The aim of this study was to assess acceptance of treatment-supportive mobile apps among men living with HIV and to determine factors influencing acceptance. The average acceptance of treatment-supportive mobile apps in the study population was moderate. Nonetheless, almost half of the participants reported high acceptance, which is consistent with previous studies in people living with HIV [28,30,36]. The moderate acceptance level could be primarily attributed to the benefits of eHealth services being offset by privacy and security concerns [56]. . Some studies using the UTAUT model showed partly similar results [57,58], while the overall acceptance in other studies was higher [40,42,45,47]. However, studies explicitly examining the acceptance of telemedicine services among patients with HIV showed comparatively low acceptance rates [27,35]. .

Considering that men with HIV often face structural barriers and participate less in routine health care [36], these findings are particularly relevant. Men, especially men who have sex with men, demonstrate lower health awareness and face stigma-related barriers, which overall leads to deficits in treatment [36,37]. The predictors of acceptance identified in this study therefore not only reveal individual factors influencing the use of eHealth, but also provide insight into how digital health tools could help address these structural deficits.

Among the sociodemographic parameters, younger age emerged as a particularly significant predictor of higher acceptance. Previous studies also found age to be an important influencing factor, though it does not necessarily determine acceptance rates. Both younger [40,43,47] and older patients [4,59] have been found to accept eHealth in previous studies. The low acceptance among older people may be explained by the fact that older people need more support when using eHealth services [60]. The age-related differences in digital usage could also be explained by lower internet access or less affinity for technology, as well as lower media literacy [61,62]. It is important to take these findings into account when developing suitable future technologies that should also be used by older people, as these eHealth technologies offer the possibility to bridge arising problems in modern health care such as the challenge of providing medical care for an aging population [4]. Other sociodemographic parameters such as level of education and place of residence were not significant predictors in this study, which aligns with the results of previous studies [39,40,45,47,48,57,58]

Including the eHealth parameters, internet anxiety in particular proved to be significant, as has been shown in previous studies [46,47,63]. This suggests that perceived limitations in technological competence and user-friendliness may negatively affect acceptance of eHealth services [64].

Previous studies have shown that patients who are well informed about their disease have better self-management and are more interested in using telemedicine services [65]. In addition, patients with higher eHealth competence, which includes eHealth literacy, also show a higher acceptance of eHealth [66]. This may explain why, in the present study, both health literacy and eHealth literacy emerged as significant predictors of acceptance.

The UTAUT core predictors EE, PE, and SI were important and significant influencing factors. This is consistent with the results of previous studies [39,40,45,47,48,57,58]. In this study, SI was found to be the strongest predictor. These results show that men living with HIV, whose social environment approves of the use of an app to support the treatment, show a higher acceptance of this app. Considering that people living with HIV are still socially excluded and discriminated against nowadays [67], it is essential to involve the environment of these individuals as well as their treating specialists for a more successful implementation of eHealth offerings like a mobile app. Other studies have found PE to be the strongest predictor of acceptance, which aligns with the results of this study [40,43,58,68]. In addition, the strong influence of EE shows that it is important to design apps that require as little effort as possible for the user.

The results of this study indicate that, among men living with HIV, promoting eHealth literacy, implementing interventions to reduce internet anxiety, and providing comprehensive information about the infection may enhance the acceptance of eHealth applications. As the recruitment strategy may have selectively favored individuals who are more interested in health-related topics and more digitally experienced, the predictors identified in this study may manifest differently in populations that are less health-conscious or less digitally proficient. Future studies should therefore specifically target these groups to generate robust and generalizable evidence on the acceptance of treatment-supportive mobile apps. Moreover, our findings indicate that designing applications tailored to both younger and older patient groups is essential to bridge the age-related barriers and to enable eHealth tools to serve as a beneficial addition to treatment.

### Limitations

Some limitations must be considered when evaluating this study. The study did not focus on a single, standardized eHealth tool, but rather referred to “treatment-supporting mobile apps” in a broader sense. While this allowed participants to draw on personal experiences and preferences, it also led to heterogeneity in the interpretation of the concept. Some respondents may have considered highly specialized adherence apps, while others may have thought of more general health monitoring tools. This variability limits the specificity of our results and should be taken into account in future studies by examining the acceptance of defined interventions.

Recruitment relied on participants’ internet access and their connection to queer community bars, HIV clinics, and social media channels. This approach was selected for a more digitally literate, socially connected, and health-engaged sample. Selection bias, therefore, needs to be considered when interpreting the results. More vulnerable groups of men living with HIV should be given special consideration in future research, as eHealth could offer particularly stigma-free and barrier-free access to health care.

Moreover, a long recruitment time for a small number of participants can cause a selection bias. Therefore, the generalizability of these findings to the broader population is limited. Only men living with HIV were included in this study, which also limits the generalizability of the results. However, the prevalence of HIV is higher among men and especially homosexual men than among women [69], so the presented results remain of high relevance for the treatment of HIV. To assess the general validity of the results, the differences between the recruited men and the male HIV-positive population in Germany must be discussed. Furthermore, participants may have been reluctant or untruthful in their responses due to the stigma associated with HIV. The risk of biased answers was reduced as much as possible by the anonymous approach of the study. The health-related variables in this study refer to self-assessed physical and mental health, rather than objective clinical indicators such as CD4 cell count, viral load, or treatment regimen. Although subjective assessments are important predictors of health behavior, the inclusion of clinical data could strengthen the validity of future analyses. The lack of such data in our study was due to its anonymous and online-based design, but future studies in clinical settings could combine both subjective and objective measures to enable a more comprehensive understanding of the determinants of acceptance. Finally, this study assesses acceptance and influencing factors but does not determine actual use. Further longitudinal studies are needed to address these limitations, particularly in populations with no experience with digital media, and to assess the true impact of the influences identified for specific applications. It is essential that women are targeted in future studies, as acceptance might differ in this cohort.

### Conclusions

This study examined the acceptance of treatment-supporting mobile apps among men with HIV using an extended UTAUT model. Overall, participants showed moderate acceptance, with nearly half expressing a high intention to use such tools. Acceptance was strongly influenced by the core predictors of UTAUT (PE, EE, and SI). Beyond the core predictors, age, internet anxiety, eHealth literacy, and health literacy related to HIV emerged as additional influencing factors.

Since acceptance is an important prerequisite for actual use and sustainable adoption of digital health solutions, the identified predictors provide valuable insights for the development and implementation of user-tailored eHealth interventions in HIV care. As SI emerged as the strongest predictor of acceptance, future implementation strategies should explicitly leverage social endorsement mechanisms. This may include the involvement of treating physicians and HIV specialists as active advocates, as well as the use of peer educators or testimonials from other men living with HIV, to enhance perceived social approval and normalize app usage within this population. By translating SI into a targeted implementation strategy, acceptance of treatment-supportive mobile apps may be further increased, thereby facilitating their successful integration into routine HIV care.

## Supplementary material

10.2196/83065 Multimedia Appendix 1 Study questionnaire.

10.2196/83065 Multimedia Appendix 2 Full hierarchical regression model of acceptance of treatment-supportive mobile apps for men living with HIV.

## Abbreviations

BI: behavioral intention
EE: effort expectancy
mHealth: mobile health
MSM: men who have sex with men
PE: performance expectancy
SI: social influence
UTAUT: Unified Theory of Acceptance and Use of Technology
