# Supplementary material for: Acceptance of Men Living With HIV Toward Treatment-Supportive Mobile Apps Using the Unified Theory of Acceptance and Use of Technology: Cross-Sectional Study
Source: JMIR Form Res. 2026 Feb 10;10:e83065. doi: 10.2196/83065 (PMC12890214; doi:10.2196/83065)
Supplement: Multimedia Appendix 1 [file formative-v10-e83065-s001.docx]

***Supplementary Materials***

Appendix S1

Acceptance of Men Living with HIV towards Treatment-Supportive Mobile Applications using the Unified Theory of Acceptance and Use of Technology: a Cross-sectional Study

Fabian Kempen^1^; Ranujan Chandrakumar^1^, B.Sc.; Stefan Esser^3^, Prof, MD; Lisa Maria Jahre^1,2^, M.Sc.; Martin Teufel^1,2^, Prof, MD; Alexander Bäuerle^1,2*^, PhD

^1^ Clinic for Psychosomatic Medicine and Psychotherapy, LVR-University Hospital, University of Duisburg-Essen, Virchowstr. 174, 45147, Essen, Germany.

^2^ Center for Translational Neuro- and Behavioral Sciences (C-TNBS), University of Duisburg-Essen, Virchowstr. 174, 45147, Essen, Germany.

^3^ Clinic of Dermatology, Department of Venerology, University Hospital Essen, Hufelandstr. 55, 45177 Essen, Germany

*Corresponding Author

Alexander Bäuerle

[alexander.baeuerle@lvr.de](mailto:alexander.baeuerle@lvr.de)

*Study questionnaire*

The questionnaire was originally administered in German and translated into English for publication of this study only.

| Variable | Items / method of assessment |
| --- | --- |
| *Sociodemographic data* |  |
| Age | Please indicate your age (in years): |
| Gender | Please indicate your gender: [single choice]   - Male - Female - Non-binary |
| Marital status | Please indicate your marital status: [single choice]   - Single - In a relationship - Married - Divorced / seperated - Other |
| Educational level | Please indicate your highest educational level: [single choice]   - No or lower secondary education / other - Higher secondary education - Higher education entrance qualification - University education |
| Occupational status | Please indicate your current occupational status: [single choice]   - Student - Non-working - Sick leave - Employed - Self-employed - Retired - Other |
| Place of residence  (population size) | Please indicate the place of residence you live in: [single choice]   - Large city (> 100,000 residents) - Medium sized city (> 20,000 residents) - Small town (> 5,000 residents) - Rural area (< 5,000 residents) |

| *Medical data* |  |
| --- | --- |
| Duration of disease | How long have you known you are infected with HIV? (in years) |
| Hospitalizations | Have you been hospitalized due to your illness?  [single choce: 0 = agree, 1 = disagree] |
| Comorbidities | Do you have any other diseases besides HIV? [multiple choice]   - No - Hepatitis C - Diabetes mellitus - Oncological cormobidities - Chronic kidney disease - Kaposi's sarcoma - Infectious diseases (e.g., esophagitis, pneumonia, fungal) - Hypercholesterolemia |
| Health literacy regarding disease | 1. Do you feel well informed about HIV infection and AIDS? 2. Do you know your T helper cell (CD4^+^) count? 3. Do you know what the T helper cell (CD4^+^) count is and what it means? 4. Do you know your viral load? 5. Do you know what viral load is and what it means? 6. Do you know the connection between T helper cell (CD4^+^) count, viral load, and the severity of your disease?   [Likert scale: 1 = “strongly disagree”, 7 = “strongly agree”] |
| Therapy satisfaction | When you think of your current HIV therapy, how satisfied are you with the following aspects:   1. Information about the disease 2. Information about the benefits and risks of treatment 3. Type of treatment 4. Side effects of treatment   [Likert scale: 1 = “not at all satisfied”, 7 = “completely satisfied”] |
| Adherence to therapy | Do you take the medication for your HIV-therapy as prescribed by your doctor? [Likert scale: 1 = “no”, 2 = “rather no”, 3 = “undecided”, 4 = “rather yes”, 5 = “yes”] |
| Physical health | How would you rate your physical health?  On a scale of 1 (very poor) to 100 (perfect) [scale 0-100] |
| Mental health | How would you rate your mental health?  On a scale of 1 (very poor) to 100 (perfect) [scale 0-100] |
| Quality of life | How would you rate your quality of life?  On a scale of 1 (very poor) to 100 (perfect) [scale 0-100] |
| *eHealth data* |  |
| eHealth literacy | Revised German version of the eHealth Literacy Scale (GR-eHEALS)  (Marsall et al. 2022) |
| Digital overload  (Bäuerle et al. 2023; Rasool et al. 2022; Schröder et al. 2023) | 1. I feel burdened by the constant availability. 2. I feel overwhelmed by unwanted messages and emails. 3. I feel uncomfortable carrying a mobile device with me all the time.   [Likert scale: 1 = “strongly disagree”, 5 = “strongly agree”] |
| Internet anxiety  (Nurtsch et al. 2024; Schröder et al. 2023; Zobeidi et al. 2023) | 1. I have concerns about using the Internet. 2. I am afraid I might make an irreversible mistake when using the Internet. 3. The Internet is something that worries me.   [Likert scale: 1 = “strongly disagree”, 5 = “strongly agree”] |
| *Acceptance (Unified Theory of Acceptance and Use of Technology, UTAUT)*  Adapted version based on the original version of the UTAUT model (Venkatesh et al. 2003) | |
| Acceptance (operationalized as Behavioral intention; BI) | 1. I would like to try an app to support my HIV treatment. 2. I would use an app to support my HIV treatment if it were offered to me. 3. I would recommend an app to support my HIV treatment to other people living with HIV. |
| Social influence (SI) | 1. People close to me would approve of me using an app to support my HIV treatment 2. My treating specialist for infectious diseases would approve of me using an app to support my HIV treatment 3. People I know who are living with HIV would approve of me using an app to support my HIV treatment |
| Performance expectancy (PE) | 1. An app to support HIV treatment could improve how I manage my infection. 2. An app to support HIV treatment could alleviate my uncertainty. 3. An app to support HIV treatment could help me improve my personal health. |
| Effort expectancy (EE) | 1. Using an app to support HIV therapy would not be an additional burden for me. 2. An app to support HIV therapy would be easy to use and understand. 3. I could incorporate an app to support HIV therapy into my everyday life.   [Likert scale: 1 = “strongly disagree”, 5 = “strongly agree”] |

**References**

Bäuerle A, Mallien C, Rassaf T, Jahre L, Rammos C, Skoda EM, et al. Determining the Acceptance of Digital Cardiac Rehabilitation and Its Influencing Factors among Patients Affected by Cardiac Diseases. J Cardiovasc Dev Dis. 2023;10(4).

Marsall M, Engelmann G, Skoda EM, Teufel M, Bäuerle A. Measuring Electronic Health Literacy: Development, Validation, and Test of Measurement Invariance of a Revised German Version of the eHealth Literacy Scale. J Med Internet Res. 2022;24(2):e28252.

Nurtsch A, Teufel M, Jahre LM, Esber A, Rausch R, Tewes M, et al. Drivers and barriers of patients' acceptance of video consultation in cancer care. Digit Health. 2024;10:20552076231222108.

Rasool T, Warraich NF, Sajid M. Examining the Impact of Technology Overload at the Workplace: A Systematic Review. SAGE Open. 2022;12(3).

Schröder J, Bäuerle A, Jahre LM, Skoda EM, Stettner M, Kleinschnitz C, et al. Acceptance, drivers, and barriers to use eHealth interventions in patients with post-COVID-19 syndrome for management of post-COVID-19 symptoms: a cross-sectional study. Ther Adv Neurol Disord. 2023;16:17562864231175730.

Venkatesh, Morris, Davis, Davis. User Acceptance of Information Technology: Toward a Unified View. MIS Quarterly. 2003;27(3).

Zobeidi T, Homayoon SB, Yazdanpanah M, Komendantova N, Warner LA. Employing the TAM in predicting the use of online learning during and beyond the COVID-19 pandemic. Front Psychol. 2023;14:1104653.
