# Supplementary material for: Acceptance of Men Living With HIV Toward Treatment-Supportive Mobile Apps Using the Unified Theory of Acceptance and Use of Technology: Cross-Sectional Study
Source: JMIR Form Res. 2026 Feb 10;10:e83065. doi: 10.2196/83065 (PMC12890214; doi:10.2196/83065)
Supplement: Multimedia Appendix 2 [file formative-v10-e83065-s002.docx]

***Supplementary Materials***

Appendix S2

Acceptance of Men Living with HIV towards Treatment-Supportive Mobile Applications using the Unified Theory of Acceptance and Use of Technology: a Cross-sectional Study

Fabian Kempen^1^; Ranujan Chandrakumar^1^, B.Sc.; Stefan Esser^3^, Prof, MD; Lisa Maria Jahre^1,2^, M.Sc.; Martin Teufel^1,2^, Prof, MD; Alexander Bäuerle^1,2*^, PhD

^1^ Clinic for Psychosomatic Medicine and Psychotherapy, LVR-University Hospital, University of Duisburg-Essen, Virchowstr. 174, 45147, Essen, Germany.

^2^ Center for Translational Neuro- and Behavioral Sciences (C-TNBS), University of Duisburg-Essen, Virchowstr. 174, 45147, Essen, Germany.

^3^ Clinic of Dermatology, Department of Venerology, University Hospital Essen, Hufelandstr. 55, 45177 Essen, Germany

*Corresponding Author

Alexander Bäuerle

[alexander.baeuerle@lvr.de](mailto:alexander.baeuerle@lvr.de)

**Table 2** Full *Hierarchical Regression Model of Acceptance of Treatment-Supportive Mobile Applications for Men Living With HIV*

| *Predictors* | *B* | β | *t* | *R^2^* | *∆R^2^* | *p* |
| --- | --- | --- | --- | --- | --- | --- |
| Intercept | .34 | .26 | -0.91 |  |  | .36 |
| **Step 1: Sociodemographic Data** |  |  |  | .131 | .131 |  |
| Age | .0 | -.01 | -0.28 |  |  | .78 |
| Educational level   (ref. No or lower secondary education/ other) |  |  |  |  |  |  |
| Higher secondary education | .23 | -.19 | 1.39 |  |  | .17 |
| Higher education entrance qualification | .08 | .07 | .54 |  |  | .59 |
| University education | -.18 | -.16 | -1.41 |  |  | .16 |
| Place of residence (Population size)  (ref. Rural area (< 5,000)) |  |  |  |  |  |  |
| Small town (> 5,000) | .48 | .41 | 1.22 |  |  | .22 |
| Medium-sized city (> 20,000) | .28 | .23 | 0.76 |  |  | .45 |
| Large city (> 100,000) | .33 | .28 | 0.99 |  |  | .33 |
| **Step 2: Medical data** |  |  |  | .149 | .018 |  |
| Duration of disease | .00 | .02 | 0.35 |  |  | .73 |
| Health literacy regarding disease | .13 | .11 | -2.08 |  |  | .04 |
| Therapy satisfaction | .12 | .10 | 1.87 |  |  | .06 |
| **Step 3: eHealth data** |  |  |  | .175 | .026 |  |
| eHealth literacy | .16 | .10 | -2.05 |  |  | .04 |
| Digital overload | .01 | .03 | 0.48 |  |  | .63 |
| Internet anxiety | -.05 | -.08 | -1.53 |  |  | .13 |
| **Step 4: UTAUT predictors** |  |  |  | .720 | .545 |  |
| EE | .38 | .32 | 5.28 |  |  | < .001 |
| PE | .23 | .21 | 3.39 |  |  | < .001 |
| SI | .48 | .40 | 6.60 |  |  | < .001 |
| *Note.* *N* = 172. In Step 2, 3, and 4 only the newly included variables are presented. *B* = Unstandardized beta. β = Standardized beta. *t* = Test statistic. *R*² = Determination coefficient. ∆*R*^2^ = Changes in *R*^2^. ref. = Reference level. EE = Effort expectancy, PE = Performance expectancy, SI = Social influence, UTAUT = Unified Theory of Acceptance and Use of Technology. | | | | | | |
